# Glyphosate-based herbicides reduce the activity and reproduction of earthworms and lead to increased soil nutrient concentrations

**DOI:** 10.1038/srep12886

**Published:** 2015-08-05

**Authors:** Mailin Gaupp-Berghausen, Martin Hofer, Boris Rewald, Johann G. Zaller

**Affiliations:** 1 Institute of Zoology, University of Natural Resources and Life Sciences Vienna, Gregor Mendel Straße 33, A-1180 Vienna, Austria; 2 Institute of Forest Ecology, University of Natural Resources and Life Sciences Vienna, Peter-Jordan Straße 82, A-1190 Vienna, Austria

## Abstract

Herbicide use is increasing worldwide both in agriculture and private gardens. However, our knowledge of potential side-effects on non-target soil organisms, even on such eminent ones as earthworms, is still very scarce. In a greenhouse experiment, we assessed the impact of the most widely used glyphosate-based herbicide Roundup on two earthworm species with different feeding strategies. We demonstrate, that the surface casting activity of vertically burrowing earthworms (*Lumbricus terrestris*) almost ceased three weeks after herbicide application, while the activity of soil dwelling earthworms (*Aporrectodea caliginosa*) was not affected. Reproduction of the soil dwellers was reduced by 56% within three months after herbicide application. Herbicide application led to increased soil concentrations of nitrate by 1592% and phosphate by 127%, pointing to potential risks for nutrient leaching into streams, lakes, or groundwater aquifers. These sizeable herbicide-induced impacts on agroecosystems are particularly worrisome because these herbicides have been globally used for decades.

During the past 50 years the human population has more than doubled, while the productive arable area has increased only by 10%^1^,^2^. As a consequence, the intensity of agricultural production has increased dramatically including the use of pesticides. Among pesticides, glyphosate-based herbicides are most widely used - hardly available data state a global usage of about 650,000 tons^3^ at sales worth about 6.5 billion US $ in 2010^4^. Glyphosate-based herbicides have been so widely used because they are very effective, acting non-selectively on plants by inhibiting the shikimic acid metabolic pathway found exclusively in plants and some microorganisms^5^. Hence, animals should theoretically not be affected by the application of glyphosate. Moreover, glyphosate is considered environmentally friendly due to its fast degradation^5^ and strong adsorption to soil particles that should reduce leaching losses from the soil profile^6^. Nevertheless, evidence that glyphosate-based herbicides can harm non-target organisms, particularly amphibians^7^,^8^, symbiotic mycorrhizal fungi or earthworms continues to mount^9^,^10^.

Earthworms constitute a majority of soil faunal biomass in many temperate agroecosystems, with up to 1000 individuals and 300 g of biomass in each square meter of land^11^. They act as ecosystem engineers^12^ by physically shredding plant litter, mineralizing it in their guts (along with soil organic matter), and enhancing soil nutrient availability through the production of up to 40 tons of casts per hectare annually^13^ that can promote plant productivity^14^,^15^,^16^. Earthworm burrowing also enhances soil root penetration and water infiltration by constructing up to 8900 km of belowground channels per hectare^17^. Thus, earthworms strongly modulate agroecosystem function and services and any factor that may harm earthworms will impact ecosystem function, including plant growth and productivity^14^,^18^,^19^.

Most studies that have examined the effects of glyphosate-based herbicides on the activity and reproduction of temperate earthworms have been conducted under laboratory conditions using compost worms (*Eisenia* species) that commonly do not inhabit agroecosystems^20^,^21^,^22^,^23^,^24^,^25^. Here, we present results of a greenhouse experiment testing the effects of a glyphosate-based herbicide on two earthworm species that are indeed frequently found in agroecosystems: the vertically burrowing anecic earthworm *Lumbricus terrestris* L. and the soil-dwelling endogeic species *Aporrectodea caliginosa* Savigny. We hypothesized that herbicide application would stimulate earthworm activity and reproduction due to the increased availability of dead plant material that earthworms can use as food source. As a knock-on effect, we expected that herbicide application via its effects on earthworms would also alter water infiltration, soil nutrient availability, and decomposition.

To test these hypotheses, we established weed communities comprising of a grass, a leguminous herb and a non-leguminous herb species commonly occurring in arable agroecosystems or garden beds. To these weed communities we added vertically burrowing or horizontally burrowing earthworm species. Eight weeks after planting, the vegetation in half of the mesocosms was treated with a lower-than-recommended dose of glyphosate-based herbicide.

## Results and Discussion

**Figure 1:**
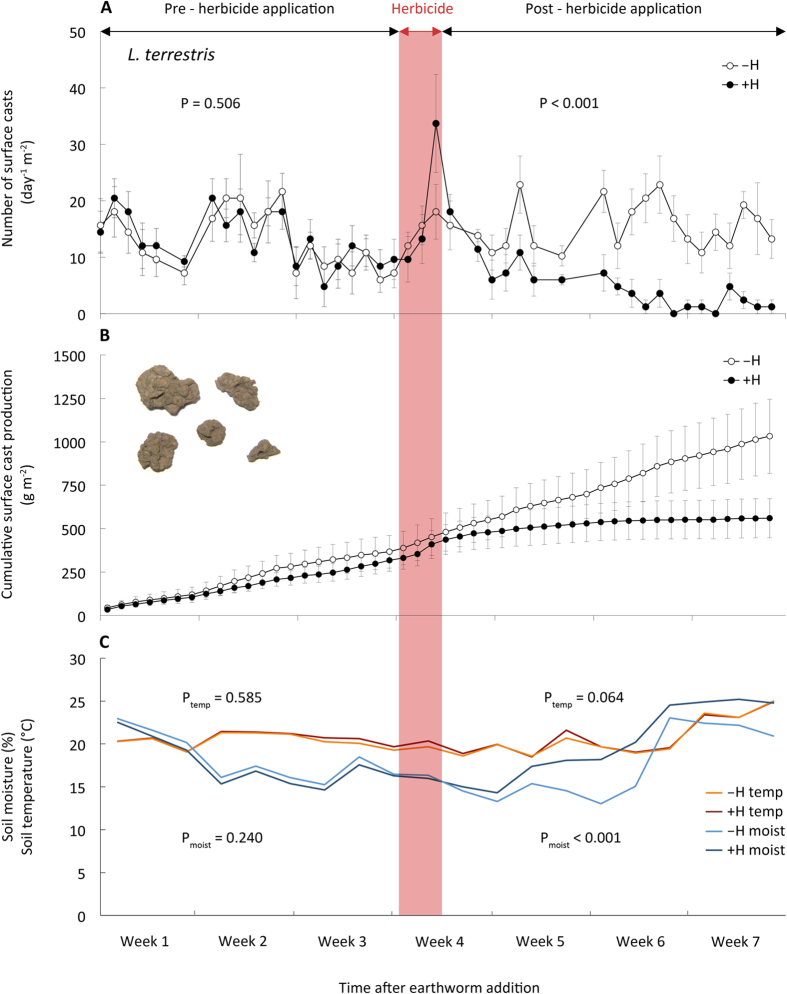
Activity of vertically burrowing earthworms before and after the herbicide application (-H, without herbicide; +H, with herbicide). (**A**) Daily surface cast production, (**B**) cumulative cast production over the course of the experiment, (**C**) time course of soil temperature (temp) and soil moisture (moist) (N = 6, mean ± SE). Red band marks period of herbicide application. P-values from two-sample Wilcoxon tests performed for the pre- and post-herbicide periods.

**Figure 2:**
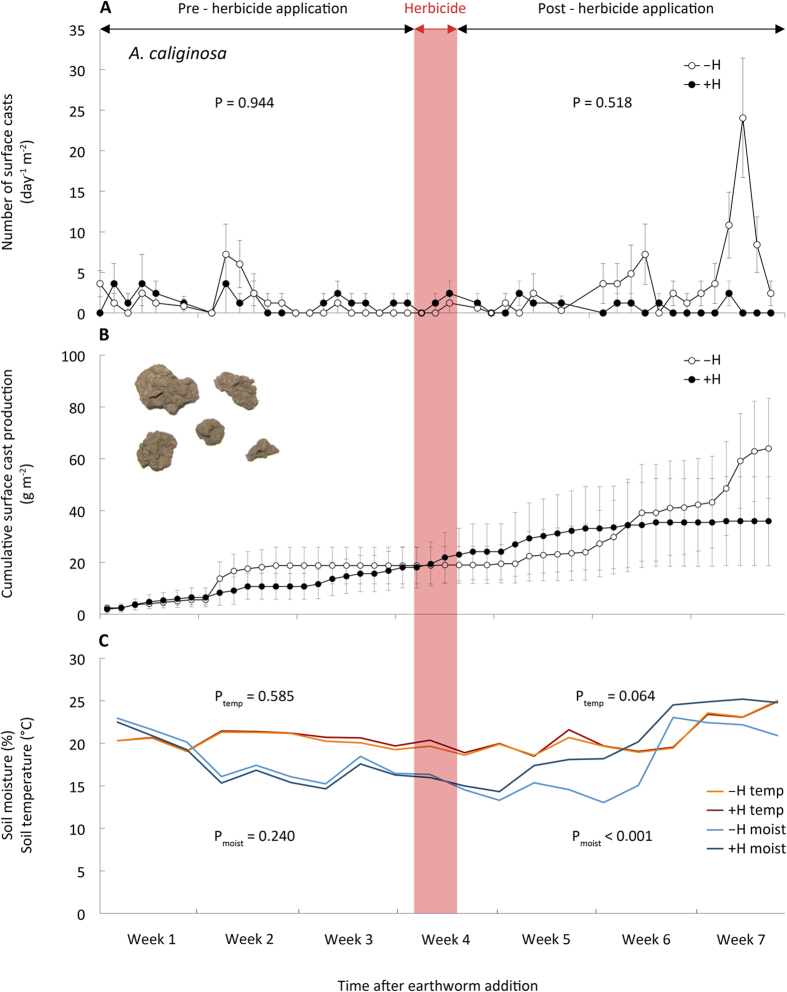
Activity of horizontally burrowing earthworms before and after the herbicide application (−H, without herbicide; +H, with herbicide). (**A**) Daily surface cast production, (**B**) cumulative cast production over the course of the experiment (N = 6, mean ± SE). Red band marks the period when herbicide was applied. P-values from two-sample Wilcoxon tests performed for the pre- and post-herbicide periods.

Herbicide application initially stimulated surface casting activity of *L. terrestris*, however the number of produced casts ceased dramatically about one week after herbicide application; in contrast the surface casting activity of this species remained nearly constant when no herbicide was applied (Fig. 1A). Not only did exposure to herbicide reduce the number of surface casts produced, it also reduced the mean mass of individual casts (546 ± 202 mg cast^−1^ vs. 1,408 ± 140 mg cast^−1^). Compared to non-herbicide treated mesocosms, cumulative cast mass produced by *L. terrestris* four weeks after herbicide application was reduced by 46% compared to untreated mesocosms (560 g m^−2^ vs. 1,032 g m^−2^; P < 0.001; Fig. 1B). Surface casting activity and cast mass production of the soil-dwelling earthworm species, *A. caliginosa*, was not affected by herbicide application (Fig. 2A,B). Monitoring surface casting activity has recently been proposed as an ecotoxicity test better related to earthworms’ ecological role than standard laboratory tests^26^. Although the studied earthworm species differ in their feeding behavior, both have been shown to cast on the soil surface when foraging for leaf litter and other organic material^27^,^28^. The peak in surface casting activity observed after herbicide application was therefore likely the consequence of an increased availability of dead leaf material. Since we provided extra food for earthworms in all treatments (i.e., dried chopped hay spread over the soil surface) which is supposed to increase surface casting activity, the further decrease in casting activity in herbicide-treated mesocosms clearly demonstrates a direct impact of the herbicide. These detrimental effects of the herbicide on earthworm activity are also surprising as soil moisture increased between 3% to 39% after herbicide application (Fig. 1C) reflecting the lack of physiologically active, transpiring plants – however, increased soil moisture commonly stimulates casting activity^13^,^29^,^30^. Another explanation for the reduced surface casting activity after herbicide treatment might also be that *L. terrestris* avoided plant residues contaminated with glyphosate on the surface. As a consequence these earthworms might have lived in deeper soil horizons and avoided surface foraging and casting. This might also suggest the – albeit not significant – higher water infiltration in mesocosms with this species when exposed to the herbicides (see below). Overall, at the end of the experiment we retrieved 93.3 ± 6.6% and 86.7 ± 9.9% of introduced numbers of *L. terrestris* and 100.0 ± 0.1% and 100.0 ± 2.6% of introduced numbers of *A. caliginosa* in –H and +H treatments, respectively.

**Figure 3:**
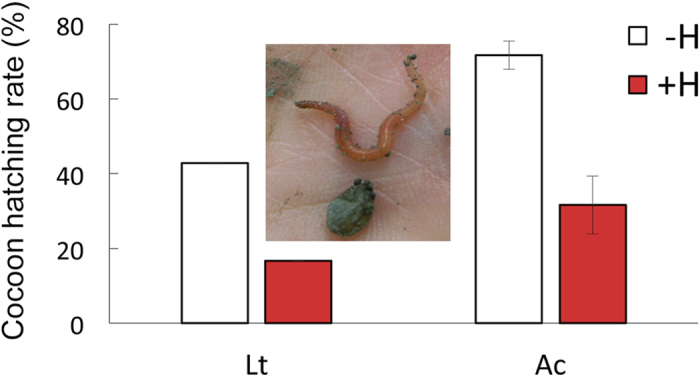
Percentage of cocoons with hatchlings of a vertically burrowing (*L. terrestris*, Lt) or a soil dwelling earthworm species (*A. caliginosa*, Ac) collected from mesocosms without (−H) and with (+H) herbicide application. (Lt: N = 1–2, Ac: N = 6, mean ± SE). Inset shows a cocoon with a freshly hatched *L. terrestris*.

Reproduction success of both earthworm species substantially decreased after herbicide application. In total we found 25 cocoons from L*. terrestris* (18 cocoons in two –H, 7 cocoons in one +H mesocosm) and 292 cocoons from *A. caliginosa* (193 cocoons in six –H, 99 cocoons in six +H mesocosms). Hatching rate, i.e., percentage of cocoons from which earthworms hatched, decreased from 43% to 17% for *L. terrestris* (no statistical test was performed because of two few replications among treatments) and from 71% to 32% for *A. caliginosa* (P < 0.001) when cocoons were collected in mesocosms without herbicide or with herbicide treatment, respectively (Fig. 3). In ecotoxicological trials in the laboratory without plants glyphosate herbicide has also been shown to decrease the growth of *A. caliginosa*^31^,^32^ and reproductive output of compost worms (*E. andrei* and *E. fetida*)^21^,^22^. However, to our knowledge, the current data are the first to demonstrate in a near-realistic setting side effects of glyphosate-based herbicides on the surface casting activity and reproduction of earthworm species that actually inhabit agroecosystems and will consequently come in contact with these pesticides.

**Figure 4:**
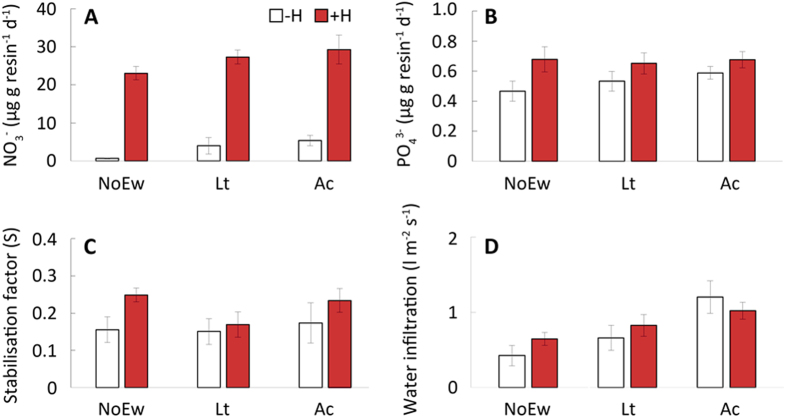
Soil parameters affected by herbicide application (–H, without herbicide; +H, with herbicide application) in response to the presence of different earthworms (NoEw, no earthworms; Lt, *L. terrestris*; Ac, *A. caliginosa*). (**A**) Plant available nitrate (NO^3−^), (**B**) plant available phosphate (PO_4_^3−^), (**C**) soil stabilisation factor, and (**D**) water infiltration rate. (N = 6, mean ± SE).

Parameters indicating important ecosystems services were also affected by herbicide treatment. After herbicide application, all plants in our mesocosms were killed within a couple of days. As a consequence plant available nitrate in the soil increased by 1592% and plant available phosphate by 127% (Fig. 4A,B), probably attributable to a decrease in nitrate and phosphate uptake by plants^33^. While, no effect of glyphosate herbicides on soil decomposition rate was found, as in previous studies^21^, the herbicide application tended to increase the stabilization factor of litter in soil suggesting a conversion from labile into more recalcitrant compounds (Fig. 4C; *26*). Herbicide application had no immediate effect on water infiltration after a simulated heavy rainfall event of 40 l m^−2^ (Fig. 4D). This was surprising as particularly vertically burrowing earthworms species such as *L. terrestris* are known to facilitate water infiltration^14^,^34^. However, the soil dwelling *A. caliginosa* that were less affected by herbicides in our study increased water infiltration (Fig. 4D, Table 1). These soil dwelling earthworms create short disconnected burrows with small diameters^35^ and have also been found to increase water infiltration rates in other studies^14^,^36^.

Because earthworms play a pivotal role in co-determining how agro- and garden ecosystems function, the observed deleterious side effects of glyphosate-based herbicide application indicate far-reaching consequences of its use in ecosystems. First, the role of earthworms as important ecological engineers in agroecosystems and gardens can be compromised^10^. Reductions in mixing of organic litter within the soil will limit long-term soil microbial activity^9^, effects of earthworms on aboveground herbivores^37^,^38^,^39^, soil nutrient cycling and availability, all of which may lead to reductions in plant productivity^40^. Second, pulses of nitrate and phosphate availability following herbicide application could increase the risk of leaching or surface runoff of these nutrients into groundwater systems or adjacent aquatic ecosystems, as long as the crop cover is not yet re-established. Obviously, official testing of potential side-effects during registration procedures failed to identify these ecologically important impacts^41^. Although productivity in many agricultural systems depends on the use of pesticides, findings from our study strongly indicate that more serious attention has to be paid testing pesticides for potential undesirable ecological side effects, especially in light of the projected doubling of global pesticide use by 2050^2^.

## Methods

### Study system and experimental setup

The experiment was performed between March and July 2013 in a greenhouse at the University of Natural Resources and Life Sciences Vienna (BOKU), Austria. We used 36 plastic pots (volume: 45 l, diameter: 42 cm, depth: 39 cm) filled with a 70 : 30 (vol/vol) mixture of soil from an arable field (soil type: Haplic Chernozem; BOKU Experimental Farm Groß-Enzersdorf) and quartz sand (grain size 1.4–2.2 mm) to create mesocosms. The substrate was homogenized using a concrete mixer, sieved (10 mm mesh size) and filled into the pots at a bulk density of 1.3 g cm^−3^. The chosen mesh size might not completely retain juvenile earthworms or cocoons already present in the field soil; however because of the thorough mixing and random distribution of the substrate among the experimental pots homogeneity across treatments can be assumed. The substrate had the following characteristics: total C = 4.41 ± 0.06 mg g^−1^, total N = 0.16 ± 0.01 mg g^−1^ (C:N ratio 27.6), K = 3.18 ± 0.12 mg g^−1^, P = 0.62 ± 0.04 mg g^−1^, and pH (CaCl_2_) = 7.45 ± 0.02 (mean ± SE). To provide an initial food source for the endogeic earthworms (see below), 1.5 g dry mass of shredded grassland hay l^−1^ soil was added to all mesocosms.

The mesocosms were planted with three types of plant species: the grass *Dactylis glomerata* L., the leguminous herb *Trifolium repens* L., and the non-leguminous herb *Taraxacum officinale* F.H.Wigg. The three species are common weeds on agricultural fields (e.g. arable land, vineyards) across Central Europe. Plants were germinated from seeds obtained from a commercial supplier specialized in wild plant populations (Rieger-Hofmann GmbH, Blaufelden-Rabholdhausen, Germany). When seedlings were 1 cm high, 17 seedlings per species were transplanted to the pots in a triangular pattern (5.5 cm between plant individuals; plant density: 51 plants mesocosms^−1^). During the experimental period, each mesocosm was irrigated equally using an automatic sprinkler system; mesocosms were placed on slats, allowing for free drainage.

Three weeks after planting, three earthworm treatments (n = 12) were established. The thirty-six mesocosms either received five specimens of adult vertically burrowing (anecic) *Lumbricus terrestris* L. (Lt) mesocosm^−1^ (25.5 ± 0.7 g mesocosm^−1^; ~183 g m^−^^2^), ten adult/sub-adult specimens of the horizontally burrowing (endogeic) *Aporrectodea caliginosa* Savigny (Ac) mesocosm^−1^ (12.09 ± 0.30 g mesocosm^−1^; ~87 g m^−^^2^), or no earthworms (NoEw). All earthworms were carefully rinsed, dried with filter paper and weighed before insertion; earthworm stockings for *L. terrestris* are in the upper range of natural abundance in temperate arable fields^42^. *A. caliginosa* was hand-collected from garden soil by one coauthor (JGZ) near the city of Eisenstadt (Burgenland, Austria), *L. terrestris* was purchased in a fishermen bait shop in Vienna. Earthworms were stored in boxes filled with the soil mixture for 5 days before transferred into mesocosms. All earthworms appeared to be in good health and buried themselves into the soil within a few minutes. Two times during the experiment 7.0 g of shredded hay were applied on the soil surface of each mesocosm, providing an additional food source. In order to prevent earthworms from escaping the mesocosm, drainage holes at the bottom of all pots were covered with garden weed fleece and the upper rim of all pots was extended with a 20 cm high, slightly outward bending barrier of transparent plastic film brushed with soft soap^39^.

Eight weeks after planting, mature plants (*D. glomerata* was about 40 cm high, *T. repens* 19 cm, *T. officinale* 31 cm) of half of the mesocosms were treated with the herbicide ‘Roundup^®^’ (treatment +H), whereas the other half of the mesocosms remained untreated (treatment –H). Each +H mesocosm was sprayed with 7.2 ml of ‘Roundup^®^ Alphée’ (glyphosate concentration 7.2 g l^−1^; Scotts Celaflor, Mainz, Germany) on two consecutive days (in sum 14.4 ml), and 10 ml of ‘Roundup^®^ Speed’ (glyphosate concentration 7.2 g l^−1^; Scotts Celaflor, Mainz, Germany) two days afterwards. In total for all applications, 176.12 ml m^−2^ of herbicide was applied which is 53% lower than the recommended plant-based application rate of 1000 plants l^−1^ for ‘Roundup^®^ Speed’ and 62% lower than the recommended dose of 800 plants l^−1^ for ‘Roundup^®^ Alphée’ (Monsanto Co., St. Louis/Missouri, USA). The manufacturer recommends both herbicides to be used as an areal application prior to planting new lawns or garden beds, however it is unclear how often these products are actually applied together. Both products are sold in ready-to-use spray bottles - according to the manual we sprayed the products homogeneously to wet all weed leaves. The manufacturer suggests repeated treatments especially for perennial weeds (www.monsanto.com).

The treatments were replicated six times in a full-factorial design: three earthworm treatments (no earthworms, Lt, Ac) and two herbicide treatments (−H, +H). To encounter influences from microclimatic gradients, the mesocosms were placed in a randomized complete block design. Soil moisture in the upper 30 cm of each mesocosm was monitored using a TDR system (6050 × 1 Trase System I; Soil moisture Equipment Corp., Santa Barbara, CA, USA); soil temperature in each mesocosm was measured at 10 cm depth (Digital thermometer az-8851; Guangzhou Orimay Electronic Co, Guangzhou, China). Air temperature and relative humidity were monitored using tinytags (Gemini Data Loggers, West Sussex, UK) at 1.5 m above the floor. Environmental conditions during the experiment: daily air temperature 22.6 ± 2.3 °C, relative humidity 58.6 ± 5.8%, soil temperature 20.2 ± 1.2 °C, and soil moisture 18.1 ± 2.6 vol% (means ± SE).

### Measurements and analyses

The measurement of earthworm activity started ten days after the introduction of earthworms by collecting freshly produced casts on the soil surface in the morning. Surface casts were collected 20 times before, three times during, and 20 times after the herbicide application; each time the casts were counted, collected, dried (50 °C, 48 h) and weighed. Cast production was expressed as number of casts produced m^−2^ day^−1^.

The water infiltration rate (l m^−2^ s^−1^) was measured two weeks after the final herbicide application by simulating a heavy rain shower of about 40 l m^−2^ (5.5 l mesocosm^−1^)^14^. The time from pouring the water onto the soil surface until the last visible water disappeared into the soil was recorded.

Plant-available nutrients in the soil were measured using ion exchange resin bags (Amberlite IRN-150; Alfa Aesar, Karlsruhe, Germany;^43^). The resin bags (7 × 7 cm nylon mesh bags, 50 μm mesh width, containing 4.5 g resin) were stored in 2 M KCl and were rinsed in deionized water. Five days prior to herbicide application one resin bag was installed per mesocosm at 10 cm depth. Excavation took place 30 days after the last herbicide application to prevent resin saturation in the rather nutrient rich soil. Collected bags were quickly rinsed in deionized water to remove adhering soil and kept refrigerated until further analysis. The solution was analyzed for NH₄^+^ and NO^3^¯ using a xMark Microplate Absorbance Spectrophotometer (BIO-RAD, Philadelphia, PA, USA) and PO₄^3^¯ using an EnSpire Multimode Plate Reader (Perkin Elmer, Walthalm, MA, USA;^44^). Plant available NH^4+^ was below detection limit and was therefore not reported.

Decomposition rate in the soil was determined using the Tea Bag Index (TBI,^45^). Therefore, two plastic tea bags containing either green tea (Lipton Unilever, USA: EAN 87 22700 05552 5) or Rooibos tea (Lipton: EAN 87 22700 18843 8) were buried at 10 cm depth in each mesocosm. Tea bags were removed 70 days after insertion (30 days after the last herbicide application). For calculating the TBI, consisting of the two parameters *k* (decomposition rate) and *S* (stabilization factor), the recommended calculated hydrolysable fractions (*H*; 0.842 g g^−1^ for green tea; 0.552 g g^−1^ for rooibos tea) were used^45^. During decomposition, parts of the labile compounds stabilize and become recalcitrant^46^. This stabilization depends on environmental factors^47^ and results in a deviation of the actual decomposed fraction (i.e. limit value) from the hydrolysable (i.e. chemically labile) fraction. Stabilisation factor S is this deviation is interpreted as the inhibiting effect of environmental conditions on the decomposition of the labile fraction^45^.

Destructive harvest of the mesocosms took place 32 days after the last herbicide application. Mesocosms were flipped over on a 2 × 2 mm mesh screen. In addition, a total number of 292 cocoons of *A. caliginosa* and totally 25 cocoons of *L. terrestris* were collected during the harvest. They were stored separated by treatment in plastic boxes (26 × 16.5 × 12 cm, length × width × depth, respectively) and mixed into the soil substrate used for the main experiment and kept in a dark basement (mean temperature 15 °C, >70% relative humidity). After 15 weeks, the number of hatched earthworms was counted and the hatchling ratio per treatment was calculated.

### Statistical analysis

Residuals of all variables were tested for homogeneity of variances and normality using the tests after Levene and Shapiro-Wilk, respectively. Assumption for normality were not fulfilled by earthworm surface casting activity and associated soil temperature and moisture. Treatment effects for parameters not fulfilling the assumption for parametric tests were analyzed using the two-sample Wilcoxon test. Effects on decomposition, soil nutrients and water infiltration rate were measured either by one-way or two-way analysis of variance (ANOVA). Each significant ANOVA result (P < 0.05) was followed by Pairwise t tests as post-hoc comparisons with sequential Bonferroni corrections to account for differences in herbicide effects within earthworm treatments. All statistical analyses were performed using R (version 3.0.1; The R Foundation for Statistical Computing; http://www.R-project.org). Values given throughout the text are means ± SE.

## Additional Information

**How to cite this article**: Gaupp-Berghausen, M. *et al.* Glyphosate-based herbicides reduce the activity and reproduction of earthworms and lead to increased soil nutrient concentrations. *Sci. Rep.* **5**, 12886; doi: 10.1038/srep12886 (2015).

## Figures and Tables

**Table 1:** Summary of two-way ANOVA results testing the effects of herbicide application and earthworms on plant available nitrate (NO_3_^−^) and phosphate (PO_4_^3−^), decomposition rate (*k*), stabilization factor (*S*), and water infiltration rate.

**Variable**	**Herbicide (H)**		**Earthworm (Ew)**		**H x Ew**	
	**F**	**P**	**F**	**P**	**F**	**P**
NO_3_¯ (μg resin^−1^ day^−1^)	176.477	**<0.001**	3.404	**0.047**	0.062	0.940
PO_4_^3^¯ (μg resin^−1^ day^−1^)	6.827	**0.014**	0.426	0.657	0.490	0.617
*k*	2.297	0.140	0.249	0.781	1.392	0.264
*S*	3.789	0.061	0.946	0.400	0.528	0.595
Infiltration (l m^−2^ s^−1^)	0.401	0.532	7.247	**0.003**	1.041	0.366

Significant effects in bold.
